# Labor and Heart Disease

**Published:** 1894-12

**Authors:** 


LABOR AND HEART-DISEASE.—Tarnier (*Journal des Sages-Femmes*, January 16, 1894) notes that in heart disease all great and sudden efforts put the patient in peril, and labor is no exception to the rule. Running up stairs, racing to catch an omnibus or train, and sexual intercourse may all cause fatal syncope. The danger of labor is not special in this sense; it is dangerous in heart disease simply because it involves much effort. Tarnier induced premature labor in a lady who was subject to advanced heart disease. Notwithstanding all precautions, she became moribund in the course of the labor. Directly she died, he turned and delivered a live child, which survived. A woman was brought into Tarnier's wards in January, 1894, in labor with advanced heart disease and asystolism; she was apparently dying. Immediately about 300 grammes of blood were withdrawn, and the symptoms of suffocation diminished. The patient grew calmer. As it was extremely advisable to bring on labor quickly, as the forceps is apt to fatigue the patient, and as, in particular, the child was dead, the basiotribe was applied and delivery effected. A few days later the mother was doing very well.—*British Medical Journal*.—*Ex.*

